# Decellularized scaffolds in regenerative medicine

**DOI:** 10.18632/oncotarget.10945

**Published:** 2016-07-29

**Authors:** Yaling Yu, Ali Alkhawaji, Yuqiang Ding, Jin Mei

**Affiliations:** ^1^ Department of Anatomy, Wenzhou Medical University, Wenzhou, China; ^2^ Institute of Bioscaffold Transplantation and Immunology, Wenzhou Medical University, Wenzhou, China; ^3^ Department of Anatomy, King Saud bin Abdulaziz University for Health Sciences, Riyadh, Saudi Arabia; ^4^ Institute of Neuroscience, Wenzhou Medical University, Wenzhou, China

**Keywords:** decellularized scaffold, extracellular matrix, regeneration, organ, *in vivo/in vitro*

## Abstract

Allogeneic organ transplantation remains the ultimate solution for end-stage organ failure. Yet, the clinical application is limited by the shortage of donor organs and the need for lifelong immunosuppression, highlighting the importance of developing effective therapeutic strategies. In the field of regenerative medicine, various regenerative technologies have lately been developed using various biomaterials to address these limitations. Decellularized scaffolds, derived mainly from various non-autologous organs, have been proved a regenerative capability *in vivo* and *in vitro* and become an emerging treatment approach. However, this regenerative capability varies between scaffolds as a result of the diversity of anatomical structure and cellular composition of organs used for decellularization. Herein, recent advances in scaffolds based on organ regeneration *in vivo* and *in vitro* are highlighted along with aspects where further investigations and analyses are needed.

## INTRODUCTION

Allogeneic organ transplantation remains the ultimate solution for end-stage organ failure; however, shortage of donor organs has resulted in extending transplantation waiting lists. Body organs are complex structures, mostly composed of various collections of tissues, made up of various extracellular matrixes and cellular components. In the field of regenerative medicine, organs are decellularized to remove cellular components to produce acellular extracellular matrix (ECM) or as known as Decellularized scaffolds. These scaffolds, since they lack cellular components and maintain ECMs, are “rejectless” when implanted, able to act as an inductive template for recellularization.

Decellularized scaffolds have become an emerging approach for treatment. The clinical use of decellularized scaffolds has been documented for applications such as blood vessels, cardiac valves and renal bladders. Even though, the current applications may be limited to tissue-level and anatomically simple organs, they ultimately provide the foundation for future complex and functioning organs regeneration.

**Figure 1:**
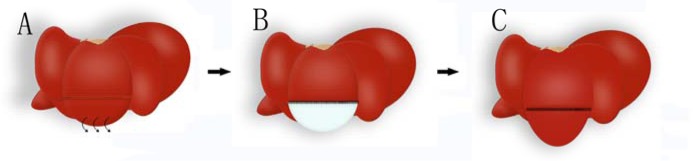
Schematic diagram of liver regeneration hypothesis using decellularized scaffolds **A.** Partial resection of one hepatic lobule is operated. **B.** The defected part is replaced with decellularized liver scaffold. **C.** Cells in the residential liver cross the suture border and regenerate on the liver scaffold.

The use of decellularized scaffolds in regenerative medicine has provided several breakthroughs recently. Despite the variability in modalities and organs used, these scaffolds have been proved a capacity to promote regeneration. *In vitro* studies, relying on bioreactors, researchers investigated the effect (role) of these scaffolds on cell proliferation and organ construction. *In vivo* implantations of decellularized scaffolds explored the effect of the scaffold on promoting angiogenesis and local regeneration (Figure 1). This rapid burgeoning of knowledge has spawned an expanding gap between research and clinical application, Herein, a review of recent advances in scaffolds based on organ regeneration *in vivo* and *in vitro* and along with areas where further investigation and analyses are needed.

## LIVER

Liver is a glandular organ, plays important roles in digestion, metabolism and homeostasis; therefore, liver is equipped with an extraordinary regenerative capability. Subsequent to hepatic tissue injury, surgical resection, poisoning, infection or necrosis of up to 80-90% of the liver, remnant hepatic tissue can rapidly regenerate to restore the original volume and weight. [1]. However, this regenerative capability may be compromised or ineffective in cases involving acute and chronic liver failure, and effective treatment for such cases largely replies on allogeneic liver transplantation. Thus constructing a portable liver by liver tissue engineering *in vitro* might be a better choice at present.

**Figure 2:**
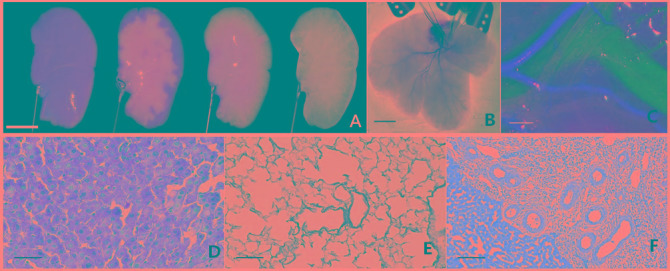
Fabrication, vascular cast, light microstructure and implantation of decellularized liver scaffolds **A.** Progressing decellularization of a single lobe of rat liver under continuous detergent perfusion. Scale bar 10mm. **B.** Decellularized whole liver scaffold with hepatic artery intact. Scale bar 20mm. **C.** Vessel corrosion casting of microstructure of the hepatic portal vein (blue), the hepatic artery (red) and the hepatic duct (transparent). Scale bar 2mm. **H.** & **E.** staining of liver matrix shows existence of blue-stained nuclei in intact liver **D.** but not in decellularized liver scaffold (**E.**). **F., H.** & **E.** staining results show the border between the liver parenchyma and implanted decellularized scaffold. Scale bar 100*μ*m.

Liver tissue engineering has made remarkable progress in recent years, providing insights into liver regeneration [2–5]. In 2010, transferable and intact acellular liver scaffolds, were developed by perfusing various chemical detergents into the portal vein in rats. These scaffolds maintained the three-dimensional structure (Figure 2) and function of the microvasculature and extracellular matrix components [3, 4]. Decellularized liver scaffolds demonstrated an ability to support efficient *in vitro* recellularization with primary hepatocytes and subsequent perfusion of cells [2, 3, 5, 6]. *In vivo* microsurgical implantation of decellularized hepatic scaffolds, involving microsurgical vascular anastomoses, showed scaffolds seeding with cells. Thrombosis formations, however, were noticed shortly post transplantation [3, 5, 7]. To address the thrombogenicity, heparin was perfused into multilayer on the inner surface of the scaffolds. [8–11] Despite the efficacy of this intervention, long-term effectiveness needs further experimentation.

## HEART

Heart has a limited regenerative capacity compared to liver. Studies have shown, cardiac stem cells in the adult heart are able to differentiate, but unable to restore functions when the heart undergoes severe pathological changes [12, 13]. Heart failure can be the end-stage of various cardiovascular diseases. Management of end-stage heart failure including pharmacological, surgical and palliative approaches cannot provide ultimate solutions.

The first decellularized cardiac scaffolds were produced from rats in 2008 [14]. These scaffolds were perfused *in vitro* with cardiomyocytes and vascular endothelial cells to mimic cardiac cell composition. Successfully, these cardiac constructs were able to perform pump function after implanting [14]. Human derived, induced pluripotent stem cells (iPSCs) were seeded into decellularized mouse hearts *in vitro*. The seeded iPSCs were able to migrate, proliferate and differentiate into functional cardiomyocytes after implanting, enabling the constructed cardiac tissues to demonstrate contractility [15]. Murine neonatal cardiac cells and human umbilical cord derived endothelial cells (HUVEC) were seeded into the left ventricle of decellularized porcine cardiac scaffolds resulted in contractive fibers formation in 50% of the injection site [16]. Yet, a thorough understanding of decellularized scaffolds effect on proliferation and differentiation of transplanted cells remains absent from the current literature.

Recently, an increasing attention has focused on mending myocardial tissue post ischemic myocardial infarctions. Bone marrow mesenchymal stem cells (MSCs) were anchored onto myocardial ischemia infarction, promoting the angiogenesis and heart repair [17]. Transplantation of stem cells improved infracted tissue condition and overall heart function [18, 19]. Considering that decellularized cardiac scaffolds offer biocompatibility and contains various cytokines, the utility of scaffold for repairing myocardial ischemia infarction area promotes the endogenous capacity of the infarcted myocardium to attenuate remodeling and improve heart function following myocardial infarction [20].

## LUNG

Clinical application of scaffold-based tracheal regeneration has been reported in the literature [21], however, regeneration of pulmonary tissue remains challenging [22]. The auto-regenerative capacity of pulmonary tissue is limited, unable to restore complete pulmonary structure and function, although, local progenitor cells can just repair the epithelial layer [23, 24]. Therefore, lung transplantation unfortunately remains the treatment for end stage lung failure [25].

Research into pulmonary tissue regeneration has been through two stages. The fundamental notion of regenerating a lung segment combines pulmonary stem cells with synthetic materials for constructing of pulmonary functional units (the alveolus), able to regenerate lung tissue. Based on such proposal, pulmonary stem cells were seeded into synthetic material *in vivo* and *in vitro*. The constructs failed to form complete pulmonary structure and function [26], possibly due to poor integration and histocompatibility and impaired respiratory function caused by post-operative infection [27].

Recently, pulmonary tissue engineering has focused on regeneration promoted by decellularized scaffold *in vivo* and *in vitro*. During decellularization, the structural proteins and relevant cytokines of extracellular matrix (ECM) are retained, whereas cellular components are removed [28, 29]. Epithelial and endothelial cells were seeded onto trachea and vessels, two independent research groups at Yale University and Harvard University found that effective gas exchange can be generated six hours later in rats with the tissue engineered lungs [30, 31].

The MSCs cultured on the pulmonary scaffold could be induced to proliferate and differentiate. There was little difference in cell proliferation and differentiation between normal pulmonary scaffold and fibrosis scaffold [32]. Whether the fibrosis alleviated and whether other cells seeded on the scaffold appear the same result still need further examination. It is something to ponder that what the outcome occurs after transplanting the engineered fibrosis lung *in vivo*.

## KIDNEY

Kidney is a parenchymal organ, composed of nearly million nephrons, uniquely arranged to eliminate body wastes and regulate water and salt balance. Due to this complexity kidney regeneration, therefore, is not an easy task [35] [36]. Nevertheless, research into cell engineering and stem cells may influence kidney regeneration [37]. A recent study indicated that adult renal progenitor cells (ARPCS) can be used to repair renal tubular damage during regeneration [38]. Renal extracellular matrix is essential for renal development and repair and signal transduction.

Porcine kidneys were successfully decellularized, proposing the possibility of using these transplantable scaffolds to construct tissue-engineered kidney clinically applicable [39]. Whole porcine kidneys were decellularized and then orthotopically *in vivo* transplanted, then prophylaxis was administrated as an anticoagulant. Inflammatory cells in the pericapsular region and thrombosis occurred due to the lack of endothelial cells [40].

**Figure 3:**
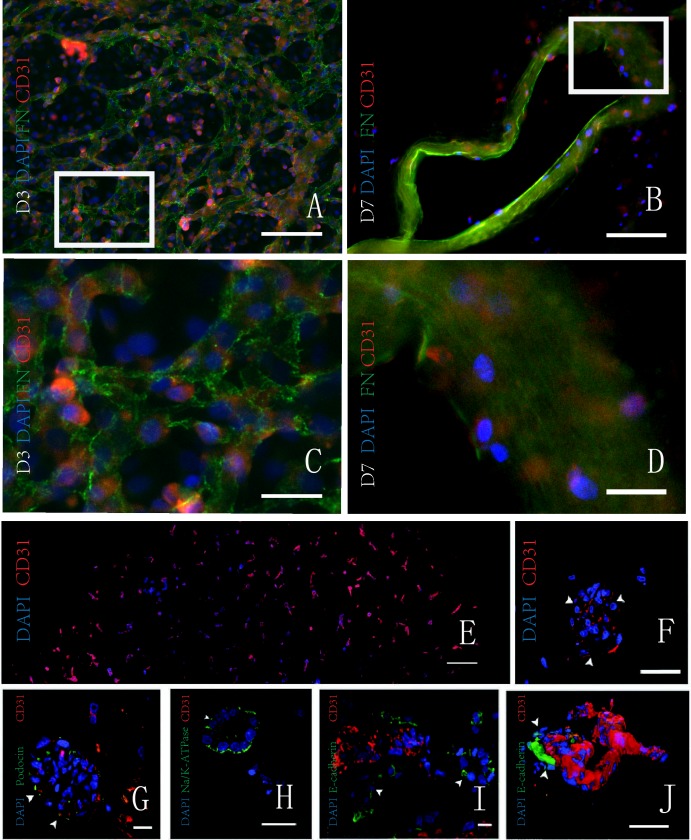
Proliferation of cells in the decellularized kidney scaffolds *in vitro.* **A. B.** Double immunofluorescence shows the scaffold and the HUVEC with fibronectin (green) and CD31 (red), respectively. On the third day, adhered HUVECs are increased. On the seventh day, HUVECs adhere to the wall of median renal vessel-like structure in the scaffolds. **C.**.**D.** The magnification pictures show the white squares in Figure. **E. F.** Fluorescence micrographs of a re-endothelialized kidney constructs. CD31 positive (red) and DAPI-positive HUVECs line the vascular tree across the entire graft cross section (image reconstruction, left) and form a monolayer to glomerular capillaries (right; white arrowheads indicate endothelial cells). **G.**-**J.** Fluorescence micrographs of re-endothelialized and re-epithelialized kidney constructs showing engraftment of podocin-expressing cells (green) and endothelial cells (CD31 positive; red) in a glomerulus (left; white arrowheads indicate Bowman's capsule and the asterisk indicates the vascular pole); engraftment of Na/K-ATPase-expressing cells (green) in a basolateral distribution in tubuli resembling proximal tubular structures with the appropriate nuclear polarity (left middle); engraftment of E-cadherin-expressing cells in tubuli resembling distal tubular structures (right middle); and a three-dimensional reconstruction of a re-endothelialized vessel leading into a glomerulus (white arrowheads indicate Bowman's capsule, and the asterisk indicates the vascular pole). T, tubule; Ptc, peritubular capillary. **A.**-**D.** Republished with permission of the Impact journals, from Jin et al. [33]; and **E.**-**J.** Reprinted from Song et al. [34] with permission from NPG, permission conveyed through Copyright Clearance Center, Inc. (For interpretation of the references to color in this figure legend, the reader is referred to the web version of this article.).

Tissue-engineered kidney was constructed using rat renal decellularized scaffolds seeded with endothelial and epithelial cells [41] *in vitro* (Figure 3). The engineered kidneys were orthotopically *in vivo* transplanted and successfully produced urine [42]. The reabsorption of partial electrolytes did not reach the level of the normal kidney, which may be associated with incomplete implantation of cells and immature endothelial cells [43]. Along with the further research, the engineered kidney *in vitro* may provide adequate kidney for patients with end-stage renal disease.

**Figure 4:**
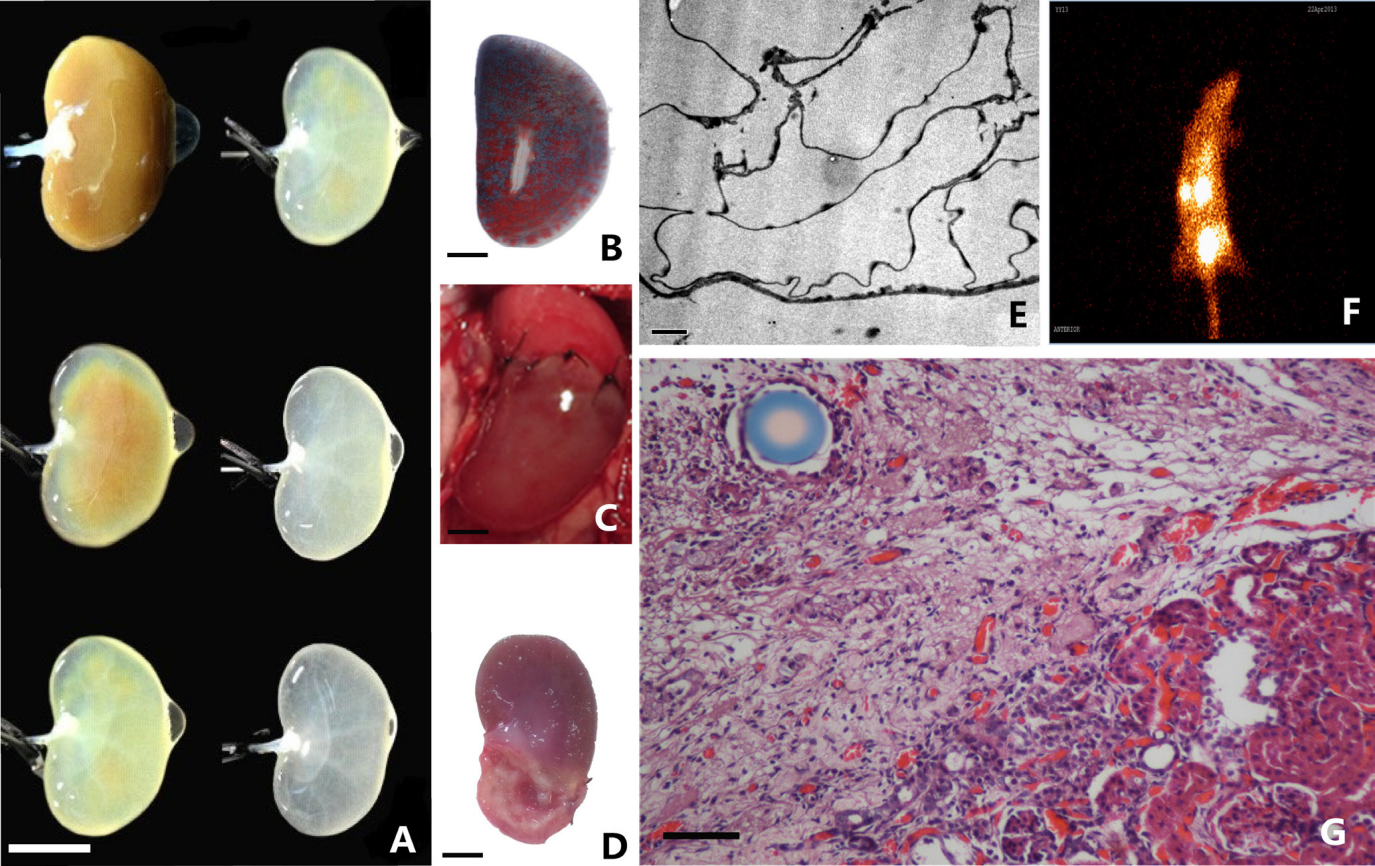
Fabrication and implantation of decellularized kidney scaffolds **A.** With continuous detergent perfusion, the rat decellularzied kidney scaffold show different gross appearance. Scale bar 10mm. **B.** Casting model of decelluarized kidney scaffolds show intact microvessels. **C.** Decellularized scaffolds was sutured to a rat underwent partial nephrectomy. **D.** Macroscopic images show longitudinal cross-sections of whole experimental kidneys observation under stereoscopic microscope. Scale bar 20mm. **E.** Electron microscopy observation shows intact extracellular matrix in decellularized kidney scaffold. Scale bar 2μm. **F.** Radionuclide scanning analysis of experimental kidneys. **G.** H&E staining shows the border between the renal parenchyma and implanted decellularized scaffold. Scale bar 100μm.

Recent findings showed that the successfully engineered renal proximal tubule had the ability of absorption, metabolism and endocrine function [44]. We successfully demonstrated that the renal decellularized scaffolds can induce regeneration of injured kidney [45] (Figure 4). The various cytokines in the scaffold may play a key role in the recovery of renal function after partial nephrectomy.

Despite the great progress has been made in recent researches, it remains difficult to reconstruct a complete functional kidney as lots of problems remain unresolved. It is essential that the engineered kidney have a complete renal function, producing urine and secretion of erythropoietin (EPO) before the regenerative kidney can be used in clinic. Further research on the stem cell biology and biological engineering is expected to open a new door for the treatment of renal damage and recovery of renal function.

## PANCREAS

Prepubescent, pancreas exhibits a vigorous auto-regeneration capability, attributed mainly to the plasticity of δcells [46] and the regulatory effect of various proteins in pancreatic extracellular matrix [47–51]. Diabetes mellitus (DM), especially type 2, demonstrate compromised insulin secretion associated with β cells dedifferentiation [52]. Re-differentiation of βcells could possibly provide cure for DM, however, there is no definitive cure in present. Regenerative medicine, with notable developments of micro-capsule technology and bioengineered niche may contribute into the advancement of islet transplantation.

Initial studies into pancreatic regeneration focused on the synthesis material such as cross-linked collagen matrix liquid scaffold [53–57]. First decellularized pancreatic scaffolds were produced from a porcine model in 2013. These scaffolds were subsequently seeded with human amniotic fluid-derived stem cells (hAFSC) and porcine islets. The scaffolds exhibited an to promote cell proliferation and maintain cellular function [58]. Pancreatic acinar cell and β cell were used to construct decellularized pancreatic scaffolds *in vitro* resulted in increased insulin level post the subcutaneous transplantation [59]. Recent studies indicated, the composed pancreas constructed by artificial three dimensional material withβcells can regulate the blood glucose level after transplanted into mouse *in vivo* [60]. The role of decellularized pancreatic scaffolds in controlling blood glucose levels remains unknown. *In vivo* transplantation success of pancreatic constructs demand proper oxygenation and re-vascularization of islet grafts [56]. Controlling and optimizing these essentials could be future research focus. (In addition, confronted with how to re-differentiate β cells that dedifferentiated in type 2 diabetes, the decellularized pancreatic scaffold may provide a solution.)

## SPINAL CORD AND BRAIN

Treatment of paralysis remains a puzzle in medicine nowadays. Paralysis occurs as a proportional resultant of damage to the central nervous system (CNS). Severe trauma or pathological conditions can lead to permanent loss of sensory and motor functions possibly due to the extremely limited auto-regenerative capacity [61, 62]. The development of tissue engineering can provide a new solution. The research strategy of regenerative medicine is the combination of biological scaffold and cell and bioactive molecules, to replace and recover the damaged tissue. The utility of scaffold has been applied in regenerating non-neural tissue with satisfactory results; however, the therapeutic potential of scaffolds for regenerating CNS tissue has not been well investigated.

First spinal scaffolds were made from rats, cellular structure, myelin and nervous process disappeared, while most of extracellular matrix structural proteins were preserved. [63] CD4^+^ and CD8^+^ cell infiltration were not obvious when it was subcutaneously embedded, positing the weakness of the immunogenicity of spinal scaffolds. Spinal scaffold, produced from rats, were combined with human umbilical cord blood mesenchymal stem cells, and then implanted into spinal cords in rats. The results showed that nerve cells migrated into the scaffold, accompanied with formation of and new myelinated axons resulting in motor function recovery. [64]

Decellularized cerebral scaffolds, derived from porcine brains, failed to maintain the original structure, but the ECM, containing glycosaminoglycans (GAGs), was successfully preserved. [65] The study suggests that the extracellular matrix could be used for cell culture due to nerve biocompatibility. Human-iPSC derived neurons can grow and mature on the matrix. Brain matrix can also be further processed into injectable hydrogel nano fiber structure. Porcine brain, spinal cord and optic nerve were decellularized using a combination of the freezing-thawing, trypsin digestion, the chemical detergents methods. The generated cross-linked scaffolds, preserved various growth factors, cultured with pc12 cells and demonstrated an ability to promote cell proliferation, migration and differentiation. The ECM from CNS appears to be more effective than bladder ECM in promoting nerve cells proliferation and differentiation. [66]

## VISCERAL ORGANS

Unlike parenchymal organs, a visceral organ is an anatomically simple, hallow organ, contains a cavity to serve as a tube or poach. Longitudinal defects to a visceral organ consequent to trauma and surgical excision as per treatment of tumors and congenital diseases can be quite difficult to treat, and may require the use of artificial synthetic materials. The utility of decellularized scaffold has been gaining attention in tissue engineering as alternative therapeutic approach for such defects. Tissue engineering research has confirmed an applicable regenerative capability of decellularized scaffolds derived from visceral organs [67, 68]. The use of decellularized scaffolds provide optimal properties, leading to elimination of cell toxicity, appropriate cell adhesion, more extensive source and avoiding the complication such as stenosis [67].

Decellularized scaffolds derived from bladder and small intestine mucosal layer, preceded the clinical applications of visceral scaffolds, have become widely used for treatment of hollow viscera defect. Bladder acellular scaffold was used for repairing bladder defects in rats in 1996 [69]. Bladder acellular scaffold, due to similarity in anatomic simplicity, were also used in reconstruction of other visceral organs, such as tympanic membrane [70], esophagus [71–73], trachea [74], larynx [75], glottis [76], thoracic wall [77], ventricular wall [78], small intestine [79], and artery [80].

Despite its applicability, the regenerative ability varies between visceral scaffolds as a result of the variability of the anatomical structure and cellular composition of organs used for decellularization. Therefore, scaffolds of different organs have different effects for regenerating an organ. Jejunum [81] [80] [79] [78] [77] scaffold is more potent promoting cell proliferation and angiogenesis compared to scaffolds of bladder [81]. However, visceral scaffolds may not be, or less, effective for parenchymal organs regeneration [82].

The process of scaffold-based regeneration of a visceral organ demands adequate blood supply to support restoration of the organ structure and components in addition to motility [67]. Scaffolds, in presence of blood supply, can promote implanted stem cells [83, 84] to enhance proliferation to functional cells, restoring functions to a some extent [85] i.e. motility may not be restored. Moreover, modified scaffold can inhibit inflammatory reactions for better integration into the recipient site [86, 87].

## SKIN

The skin is the largest organ, covering the entire body and providing protection. Various appendages within the skin function to equip the skin with sensation, lubrication contractility and thermoregulation potentials, ultimately to maintain the internal environment. In addition, the skin serves as a physical defensive barrier against external hazards. Any defect to this barrier entails a rapid and efficient repairing, therefore, the abundance of stem cell in the skin empowers to a strong regeneration capability [88]. Repairing of major skin loss beyond this regenerative capability may require the transfer of autologous tissue. However, the transfer of autologous tissue, in certain situations, may be unavailable or cannot fill the defect, emphasizing the need of a backup approach to prevent greater mortality. Tissue engineered skin, first biomaterial used clinically, has been increasingly used to address this need.

The development of bioengineered products of different skin layers - including the tissue engineered epidermis, dermis and composite skin - has provided innovative tools for clinical applications. Cultured epithelial autograft (CEA), an approach to obtain epidermal grafts, has been used in repairing of major burns. Tissue-engineered epidermal cells, prepared by culturing autologous human epidermal keratinocytes *in vitro*, was grafted for repairing burn wounds in two patients. [89] However, absence of dermis layer and wound contracture and may lead poor cells adhesion and subsequent survival. Moreover, scar contracture and blistering, in later stages, have been reported. Artificial skin, developed through extensive experimentation, comprised of a layer of Silastic (epidemis) and a porous bovine collagen-chondroitn 6-sulfate (dermis), was physiologically used for repairing extensive burn injuries, constituted 50—95% of body surface area. [90] Compared to the engineered epidermis, the skin scaffolds has the ability of promoting migration of fibroblasts and angiogenesis and providing optimal mechanical and physiochemical properties necessary for healing.

In late 1990s, remarkable progress has been made in the clinical application of bioengineered products with the use of human derived products in treating burned patients [91, 92]. Advances in of acellular scaffold technology have led to improved mechanical and biological properties of acellular dermal matrix (ADM). However, the porcine dermal acellular scaffolds remain widely used in clinical application [93, 94]. More recently, stem cells research has further enhanced the progress of tissue engineered skin. Bone marrow [95] and adipose [96] derived stem cells was induced to differentiate and were implanted in the dermal acellular matrix. The composite matrix has superior ability of promoting wound healing than the pure acellular dermal matrix. The property of engineered skin with appendages should be optimized in the future

## CONCLUSIONS

Organs show huge differences in the regeneration capability (Table 1), due to the structure of various organs has individual specificity. The regenerative mechanisms of various organs differ from each other and therefore, strategies in organ regeneration based on the decellularized scaffold should be diversified. Different clinical needs reveal different research emphasis. New heart, liver and kidney are needed for patients with cardiac, hepatic or renal failure. Tissue engineered organs created from decellularized scaffold, bioreactors and seeding cells can meet this demand. For local damage and tumor within organs, decellularized scaffolds can be used as patches to repair defects. Furthermore, some chemical techniques improving regeneration become very necessary, such as modification via heparinization and kinds of growth factor. Drug-loading methods widely applied to artificial scaffolds can be introduced into decellularized scaffolds sooner. In addition, decellularized scaffolds have been widely reported to improve tissues and organs regeneration. However, related mechanisms are poorly understood. In-depth revelation on internal mechanisms will lead the development of this research field.

**Table 1:** Recent advances in scaffold based organ regeneration research *in vivo* and *in vitro*

organ	In vitro	In vivo	reference
**kidney**	Construct engineered renal proximal tubule Promote cell proliferation and differentiation such as iPS with scaffold Construct engineered kidney by precursor or differentiated cell	Renal regeneration mediated by decellularized kidney scaffold Production of urine with implanted tissue engineered kidney	[36, 38, 40, 41, 93, 94]
**Heart**	Induce precursor cells to differentiate into cardiomyocytes with decellularized scaffold The function of beat of biological engineered heart	Promotion regeneration of myocardium in the area of myocardial ischemia infarction	[14–17]
**Liver**	The support of scaffold for the primary liver cell or various cell that can be induced into hepatocytes	Establish a vascular network rapidly and recover partial compensate function	[2, 3, 5–10]
**Pancreas**	The promotion of pancreatic islet cell proliferation and support function Construction of engineered pancreas	Increase the expression of insulin gene by subcutaneous transplantation of engineered pancreas Regulation of blood glucose levels with engineered islet transplanted into mice with type 1 diabetic	[11, 49, 51, 52, 54–56, 95]
**CNS**	Preservation most matrix of spinal cord acellular scaffold. The modified matrix improve the mechanical property and promote the cell proliferation, migration and differentiation	The transplantation of combined scaffold with HUCB-MSCs can form the neo-axons with myelin sheath, and the recovery of motor function in rat	[60–62, 96]
**Bladder**		The bladder acellular scaffold promoted regeneration of epithelial cells, smooth muscle cells, vessels and nerve, which can be enhanced by stem cells	[65, 79, 80, 97]
**Esophagus**	Promotion of expression of marker protein by mucosal epithelial cells with scaffold, being suitable for cell survival and inhibiting apoptosis	The cover of esophageal sauamous epithelium, the regeneration of collagen fiber and inherent muscle layer	[75, 98–100]
**Trachea**	The co-culture of scaffold and cell can promote the proliferation of lung epithelium and endothelial cells	The appearance of ciliary epithelium and angiogenesis with tracheal transplantation	[101–105]
**Stomach**		The regeneration of proton pump and thin layer of muscle with gastric patch	[81]
**Intestinal tract**		Regeneration of intestinal tract, the cover with small intestine mucous and the appearance of muscle and nerve layer.	[106–108]
**Skin**	Engineered dermis seeded with fibroblasts, endothelial cell can promote cell proliferation and adhesion	The engineered dermis, the acellular dermal matrix (AlloDerm) can be applied in burned wound healing, breast reconstruction and transplantation of combined stem cell with dermal matrix for abdominal wall hernia.	[85–92]
